# Effect of Straw and Straw Biochar on the Community Structure and Diversity of Ammonia-oxidizing Bacteria and Archaea in Rice-wheat Rotation Ecosystems

**DOI:** 10.1038/s41598-019-45877-7

**Published:** 2019-06-27

**Authors:** Hanlin Zhang, Huifeng Sun, Sheng Zhou, Naling Bai, Xianqing Zheng, Shuangxi Li, Juanqin Zhang, Weiguang Lv

**Affiliations:** 1 Eco-environmental Protection Institute, Shanghai Academy of Agricultural Science, Shanghai, 201403 China; 2 Agricultural Environment and Farmland Conservation Experiment Station of Ministry Agriculture, Shanghai, 201403 China; 3 Shanghai Key Laboratory of Horticultural Technology, Shanghai, 201403 China; 4 Shanghai Engineering Research Center of Low-carbon Agriculture, Shanghai, 201415 China

**Keywords:** Soil microbiology, Agroecology

## Abstract

Ammonia oxidation is the first and rate-limiting step of nitrification, driven by ammonia-oxidizing bacteria (AOB) and ammonia-oxidizing archaea (AOA). Straw and straw biochar retention are the popular ways to utilize the agricultural by-products in China, but their long-term effects on AOB and AOA still remain poorly understood. Based on a 7-year plot experiment, which had 4 fertilization regimes: no fertilizer (CK), regular fertilization (RT), straw retention (SR) and straw biochar retention (SB), the abundance and the composition of AOB and AOA was investigated before both the harvest of rice and wheat season by quantitative PCR and 454 high-throughput pyrosequencing, respectively. (1) Compared to RT, straw and straw biochar increased AOB abundance and diversity significantly only in wheat season (*P* < 0.05), and they both ranked as SB > SR > RT. Among fertilized treatments, a significant difference between SR and RT was found in AOB community composition of the winter season (*R* value = 0.58, *P* value = 0.02); (2) In contrast, AOA was almost not responsive to organic addition, except the significant enhancement of abundance by biochar in wheat season; (3) After straw and straw biochar addition, soil potential nitrification rates (PNR) was positive correlated to AOB abundance in both rice and wheat season (*P* < 0.01), not to AOA abundance (*P* = 0.211 and 0.068, respectively). This study provides scientific support for the potential of straw utilization to improve nitrification in rice-wheat rotation system with respect to soil ammonia oxidation microorganism.

## Introduction

The crucial and rate-limiting step of nitrification, ammonia oxidation, converting ammonia to nitrite, is controlled by both ammonia-oxidizing bacteria (AOB) and ammonia oxidizing archaea (AOA)^1,2^. AOA abundance (amoA gene copy numbers) usually outnumbers AOB abundance in terrestrial systems, especially in agricultural ecosystems^3,4^. However, the contributions of AOB and AOA in nitrification vary depending on environmental conditions^5,6^. Therefore, studies of AOB and AOA community abundance and diversity, and their links with the ecological function are of great importance.

Soil pH was commonly considered as a key driving environmental factor influencing AOB and AOA community. AOA appeared to be more active in acidic environment, while AOB contributed more to nitrification in alkaline and neutral environment^5,7^. Soil nutrient level was another direct factor to affect AOB and AOA community. AOB seemed to be predominant under high ammonia concentration but had little response to organic matter input^8,9^, while AOA may play an important role under limited available nutrient levels^10^. In addition to soil properties, both short-terms and long-terms chemical fertilization can increase AOB abundance and alter its community composition, but almost have no impact on AOA^11,12^. Organic inputs such as organic fertilizer, straw, biochar affect AOB community significantly, but maybe because of the mixtrophic character of AOA, have no clear effect pattern with AOA community^9,13,14^. Besides, soil types and texture, vegetation type, temperature and precipitation can affect the composition of AOB and AOA community^15,16^. Although extensive researches had explored how the multiple environmental factors affect AOB and AOA communities, the effects of organic inputs on AOB and AOA community diversity and their relative contributions to nitrification are still not clear.

Crop straw is the largest renewable resource on earth. However, it is often burned after harvest, resulting in loss of nutrients and environmental pollution. With current advocate for ecological agriculture in China, straw and straw biochar returning to fields have become important modes of reutilization of agricultural by-products. Straw and straw biochar could impact the soil microbial community directly by their own and indirectly through changing soil physico-chemical properties. Hai^17^ reported that fertilizer combined with straw could increase AOB number, and AOA abundance was higher than AOB abundance, but independent of fertilizer regimes. However, the incubation experiment of Wua^18^ showed that adding wheat straw did not change the AOB and AOA abundance significantly. Similarly, the effect of biochar addition on ammonia oxidation microbial community was also not consistent. The results of Bi^19^ in vegetable soil demonstrated N fertilizer+ biochar increased abundance of AOB rather than which of AOA. He^20^ found that the biochar raised gene copies of AOB and AOA significantly in oxisols but had little effect in cambosols. Therefore, more studies should be employed for the establishment of more efficient use of straw to conserve the abundance and diversity of AOB and AOA community.

Rice-wheat rotation was applied widely in eastern China, and it is a representative wet-dry alternative system, resulting in an oxic/anoxic soil environment, which can also impact AOB and AOA community abundance and diversity significantly^21,22^. However, most of the researches determining the composition of ammonia oxidation microbial community were based on sampling in one season. Sampling in both rice and wheat seasons may give us a better understanding of the changes in the composition of ammonia oxidation microbial community in the whole rotation systems.

In this study, soil samples were collected before the harvest of rice and wheat season. Based on long-term plot experiment of straw and straw biochar, 454 high-throughput pyrosequencing and real-time quantitative PCR (qPCR) approaches were employed to investigate the abundances and community composition of AOB and AOA in the rice-wheat rotation ecosystems. The aims of this research were under the effects of straw and straw biochar (1) to identify the abundance and community composition shifts of AOA and AOB in rice and wheat season; (2) to detect the relative contribution of AOA and AOB to nitrification seasonally; (3) to provide scientific support for the establishment of a straw utilization technique to efficiently conserve the diversities of AOB and AOA communities.

## Materials and Methods

### Site description and experimental design

The six years experiment is located at Shanghai engineering research center of low-carbon agriculture, Shanghai, China (31°53′N, 121°23′E). The annual average temperature of this area is 15.7 degrees and the annual precipitation is 1162.0 mm; there are 225 d of frost free period in a year. The soil type is sandy loam.

The experiment started from rice season of 2011, and ended in wheat season of 2017. There were 4 treatments: regular treatment (RT), straw retention treatment (SR), straw biochar treatment (SB) and no fertilizer control (CK). Each treatment had 3 replicates, and all the replicates were arranged in random block design. Every replicate field was 60 m^2^, and separated by cement ridge and Impermeable membrane. All 4 treatments were treated with the same amount of nitrogen (N), phosphorus (P) and potassium (K) annually (225, 112.5 and 255 kg·ha^−1^ for rice season; 180, 90 and 204 kg·ha^−1^ for wheat season, respectively). The amount of applied N, P, K input was set according to the average application of rice-wheat rotation systems in Shanghai. The fertilizers used in the experiment were urea, Ca(H_2_PO_4_)_2_ and K_2_SO_4_. In SR treatment, the half amount of straw were all directly returned to the field; in SB treatment, the half amount of straw (rice straw applied in the wheat season and wheat straw applied in the rice season) was processed into biochar and returned to the field. Preparation of biochar was anaerobically performed at 500 °C for 6 h in a vertical charcoal furnace (ECO-5000, Wuneng Environment Co., Ltd., Zhejiang, China). N, P, K of straw and straw biochar was included in the total amount of applied fertilizer. The average N, P, K content of rice and wheat straw were 0.33 ± 0.02%, 0.19 ± 0.01%, 1.35 ± 0.08% and 0.38 ± 0.03%, 0.24 ± 0.01%, 1.84 ± 0.10%, respectively. The average N, P, K content of straw biochar in rice and wheat season were 0.84 ± 0.05%, 0.52 ± 0.04%, 5.30 ± 0.32% and 1.71 ± 0.09%, 0.64 ± 0.04%, 4.72 ± 0.21%, respectively.

### Soil sampling and analysis

Soil samples were collected in Nov. 4, 2016 and May 15, 2017, before the harvest of rice and wheat, respectively. Soil samples (0–20 cm) taken randomly from 5 replicate points of each block and mixed into one, resulting in 24 samples totally. The soil samples were stored in polyethylene bags and taken back to lab immediately. One portion of the samples was freeze-dried for soil properties determination, and the others were stored at −20 °C for DNA extraction and microbial analysis.

Soil total N was determined by Kjeldahl method; exchangeable NH_4_^+^-N and NO_3_^−^-N were extracted with 2M KCl (soil/water ratio was 1:5) and measured by SmartChem Intelligent automatic chemical analyzer (Alliance Instruments, France); soil pH was determined by potentiometry (soil -water ratio was 2.5:1); soil organic C was determined by dichromate oxidation.

Potential nitrification rate (PNR) was determined according to Kurola *et al*.^23^. Five g of soil sample were put into a 50 mL tube, mixed with 20 mL of phosphate buffered saline solution with 1 mM (NH_4_)_2_SO_4_. The tubes were shaken at the speed of 180 rpm in the dark under 25 °C for 24 h, 10 mg·L^−1^of potassium chlorate was added to inhibit nitrite oxidation. Five mL of 2M KCl were added to extract NO_2_-N after shaking (170 rpm for 24 h). The NO_2_-N concentrations in the supernatant were determined by Griess reagent colorimetric method (measured absorption at 545 nm). PNR was expressed by the unit as μg (NO_2_-N)·g^−1^·h^−1^ dry soil.

### DNA extraction and quantitative PCR analysis

Soil DNA was extracted using MoBio PowerSoil® DNA Isolation Kit (12888) according to the manufacturer’s instruction. The extracted DNA concentration and quality were determined by spectrophotometer (RS232G, Eppendorf, Germany).

The AOB and AOA gene copy numbers were determined using Applied Biosystems ABI StepOnePlus Real-Time PCR instrument. The primer pairs for AOB and AOA were amoA1F (5′-GGGGTTTCTACTGGTGGT-3′)/amoA2R (5′-CCCCTCKGSAAAGCCTTCTTC-3′)^24^ and ArchamoAF (5′-STAATGGTCTGGCTTAGACG-3′)/Arch-amoAR (5′-GCGGCCATCCATCTGTATGT-3′)^25^, respectively. The qPCR amplifications were carried out in a total volume of 20 μL, containing 10 μl of SYBR real-time PCR premix (Takara Biotechnology, Dalian, China), 4 μM of respective primer and 1 μL (approximately 20 ng) DNA template. Amplifications were conducted with the following thermal conditions: 6 min initial denaturation at 95 °C, followed by 45 cycles of 95 °C for 15 s, and 60 °C for 45 s. The qPCR was run in duplicates, and the specificity and efficiency of amplification was confirmed by melting curve analysis after every run. Standard curves were generated by a serial dilution of purified plasmid DNA harboring amoA genes (Bacteria and Archaea).

### PCR amplification and Pyrosequencing

AOB and AOA amoA genes were amplified with the primer pairs of amoA-1F/amoA-2R and Arch-amoAF/Arch-amoAR, respectively. The PCR amplifications were carried out in a total volume of 25 μL, containing 40 ng of DNA template, 1.0 μL each primer (10 μM), 0.25 μL Q5 high-fidelity DNA polymerase, 5.0 μL 5* High GC Buffer, 5.0 μL 5*Reaction Buffer, 0.5 μL dNTP (10 mM). Amplifications were conducted with the following thermal conditions: 30 s initial denaturation at 98 °C, followed by 27 cycles of denaturation at 98 °C for 15 s, annealing at 58 °C/55 °C (AOB/AOA) for 30 s, extension at 72 °C for 30 or 45 s (for amoA genes of AOB and AOA, respectively), and a final extension at 72 °C for 7 min. The quantity and quality of the PCR products was detected using NanoDrop ND-1000 UV-Vis spectrophotometer. After successful amplifications, the PCR products were pooled in equimolar concentrations of 10 ng·μl^−1^ for pyrosequencing.

The pyrosequencing was performed on a Roche massively parallel 454 GS-FLX Titanium sequencer (Roche 454 Life Sciences, Branford, CT, USA) at Shanghai Personal Biotechnology Co., Ltd. (Shanghai, China). Raw sequences were going through quality control and assigned on unique 10-bp barcodes using QIIME software (version 1.7.0)^26^. Then the remaining sequences were clustered into operational taxonomic units (OTUs) based on the level of 97% sequence similarity.

### Statistical analysis

One-way analyses of variance (ANOVA) with Turkey’s HSD tests were performed to discriminate significant differences between treatments using SPSS 19.0. The AOB and AOA richness and diversity indices (Chao1, Abundance-based and Coverage Estimator ACE, Shannon and Simpson) were estimated using Mothur (version v.1.30.1). Venn diagrams were generated to show the shared and unique genera among 4 treatments by Venn Diagram package of R software. Principal components analysis (PCA) based on the OTU composition was carried out to investigate the differences in the composition of microbial community using R software (Version 3.0.2). Analysis of similarity (ANOSIM) was applied to examine the effects’ significance of fertilization regimes on the composition of AOA and AOB using QIIME. Pearson correlation was performed to test the associations between the amoA gene abundances (AOA, AOB) and the environmental variables.

## Results

### Soil properties characteristics and crop yield

**Figure 1:**
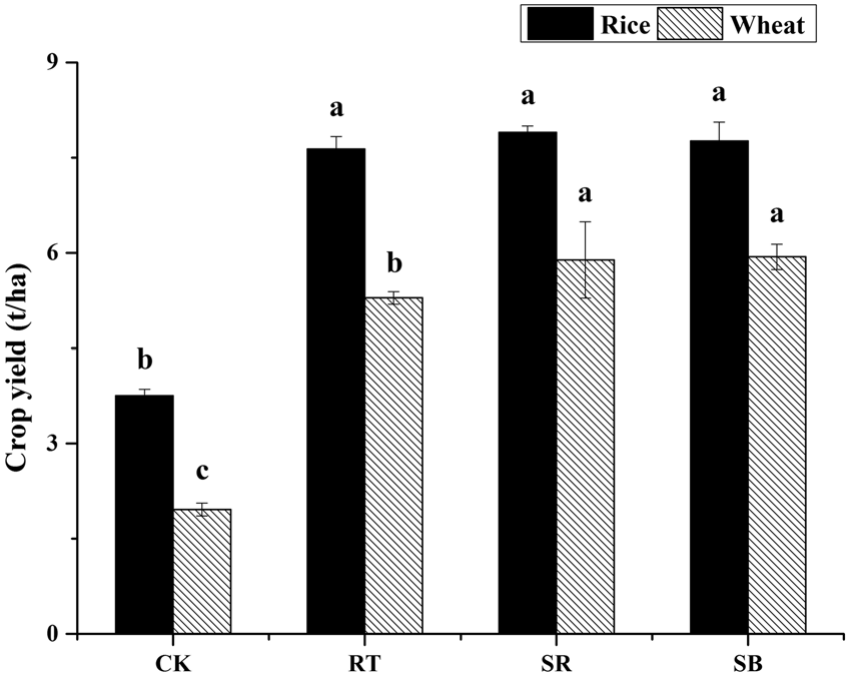
Crop yields of different treatments in rice and wheat season. Different letters above the columns indicate significant differences at *P* < 0.05.

The soil properties were affected significantly by fertilization regimes both in rice and wheat seasons (Table 1). In the harvest stage of rice season, compared to RT, SR and SB increased organic C (SOC), total N (TN) and exchangeable NH_4_^+^-N, and deceased pH and bulk density of soil significantly. There was no significant difference in NO_3_^−^-N among 4 soils. SOC, pH and exchangeable NH_4_^+^-N under SB were higher than under SR (*P* < 0.05). SR had the lowest C/N (8.5) among 4 treatments. In wheat season, there was no significant difference in all measured properties between SR and SB. Compared to RT and CK, SR and SB enhanced SOC, TN, C/N, and reduced pH, soil bulk density significantly. There was no significant difference in exchangeable NH_4_^+^-N and NO_3_^−^-N among RT, SR and SB. From rice season to wheat season, NO_3_^−^-N rocketed, while exchangeable NH_4_^+^-N declined significantly in all treatments. Fertilization increased the crop yield of rice and wheat significantly (Fig. 1). There was no significant difference among 3 fertilization treatments in rice season. However, the wheat yield of SR and SB were higher than RT by 7.3% and 8.1%, respectively.

**Table 1:** Soil properties of rice and wheat season after six years of treatments.

Season	Treatment	pH	Bulk density g·cm^−3^	SOC g·kg^−1^	TN g·kg^−1^	NO_3_^—^N mg·kg^−1^	NH_4_^+^-N mg·kg^−1^	C/N
Rice	CK	8.43 ± 0.11b	1.41 ± 0.05a	8.8 ± 0.59c	0.94 ± 0.09c	1.9 ± 0.1c	5.4 ± 0.2d	9.4 ± 0.3a
	RT	8.32 ± 0.08b	1.47 ± 0.07a	9.3 ± 0.50c	1.05 ± 0.07b	2.1 ± 0.1c	6.1 ± 0.1c	8.9 ± 0.2ab
	SR	8.03 ± 0.05d	1.31 ± 0.04b	10.5 ± 0.57b	1.24 ± 0.11a	2.3 ± 0.2c	7.6 ± 0.2b	8.5 ± 0.3c
	SB	8.17 ± 0.06c	1.33 ± 0.03b	12.1 ± 0.61a	1.31 ± 0.13a	2.4 ± 0.1c	11.6 ± 0.4a	9.2 ± 0.2b
Wheat	CK	8.60 ± 0.09a	1.44 ± 0.05a	7.5 ± 0.45d	0.87 ± 0.07c	14.2 ± 0.69b	3.1 ± 0.1 f	8.6 ± 0.2c
	RT	8.51 ± 0.10a	1.49 ± 0.08a	7.7 ± 0.47d	0.91 ± 0.08c	19.6 ± 0.74a	4.3 ± 0.2e	8.5 ± 0.3c
	SR	8.11 ± 0.04c	1.31 ± 0.03b	10.2 ± 0.42b	1.04 ± 0.09b	19.5 ± 0.61a	4.4 ± 0.1e	9.8 ± 0.3a
	SB	8.21 ± 0.06c	1.34 ± 0.05b	10.7 ± 0.51b	1.08 ± 0.08b	20.4 ± 0.52a	4.3 ± 0.2e	9.9 ± 0.4a

Abbreviations: SOC, soil organic C; TN, total nitrogen.

Dates in the table are Mean ± SE; Different letters in the same column indicate a significant difference (*p* < 0.05).

### Abundance of AOA and AOB

**Figure 2:**
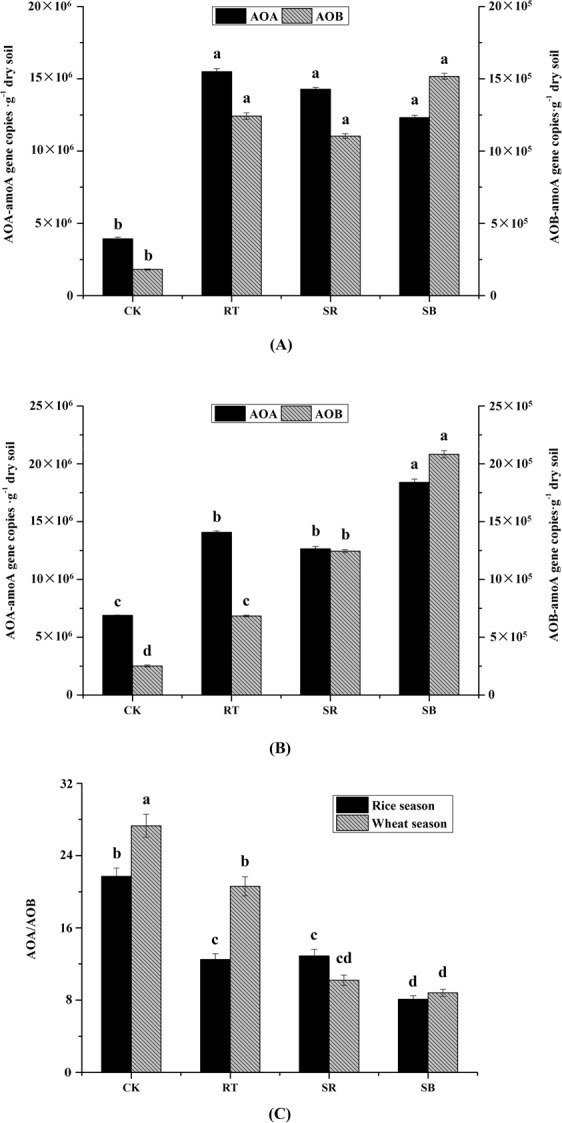
AOA and AOB amoA gene copy numbers (**A** for rice season, **B** for wheat season) and AOA/AOB (**C**) in different treatments. Different letters above the columns indicate significant differences at *P* < 0.05.

Both AOA and AOB amoA gene copy numbers were higher in fertilized soil than CK in rice and wheat seasons (*P* < 0.01) (Fig. 2). AOA gene copy numbers were not significantly different among RT, SR and SB in the rice season, but they were higher in SB than RT and SR in the wheat season (*P* < 0.01). There was also no significant difference of AOB gene copy numbers among RT, SR and SB in the rice season, but there were significant differences among RT, SR and SB (*P* < 0.01) in the wheat season, and they ranked as SB > SR > RT. AOA gene copy numbers in the whole year ranged from 3.93 × 10^6^ gene copy•g^−1^ dry soil to 1.84 × 10^7^ gene copy•g^−1^ dry soil, and AOB gene copy numbers ranged from 1.81 × 10^5^ gene copy•g^−1^ dry soil to 2.08 × 10^6^ gene copy•g^−1^ dry soil. AOA gene copy numbers were always higher than AOB gene copy numbers. AOA/AOB ratio varied from 8.1 to 21.7 and 8.8 to 27.3 in rice and wheat season, respectively. SB and CK had the lowest and highest AOA/AOB ratio in both rice and wheat seasons. AOA/AOB ratio under SR was higher than under RT in the rice season but there was no significant difference in the wheat season (*P* < 0.01).

### Alpha-diversity of AOA and AOB

Richness and diversity of AOA and AOB under different fertilization regimes were reported in Table 2. Chao1 and abundance based coverage estimation (ACE) were employed as richness indices. Simpson and Shannon indices, estimated by richness and species abundances, were used as diversity indices. For all the indices estimated, CK had the lowest richness and diversity for AOA and AOB in the whole year. The three different fertilization regimes had not affected on α-diversity of AOA community both in rice and wheat season. According to richness and diversity indices of AOB, there were significant differences among SB, SR and RT treatments in the wheat season, and these treatments ranked as SB > SR > RT (*P* < 0.05); while there was no significant differences in the rice season.

**Table 2:** Richness and diversity indices of AOA and AOB in rice and wheat season.

Season		Treatment	Chao1	ACE	Simpson	Shannon
Rice	AOA	CK	510.65 ± 15.55b	531.79 ± 10.11b	0.73 ± 0.01b	3.92 ± 0.21b
		RT	588.84 ± 20.61a	613.36 ± 12.41a	0.85 ± 0.02a	4.38 ± 0.18a
		SR	600.94 ± 21.94a	595.36 ± 13.05a	0.80 ± 0.01a	4.27 ± 0.14a
		SB	632.36 ± 19.74a	648.60 ± 20.84a	0.86 ± 0.01a	4.34 ± 0.17a
	AOB	CK	85.50 ± 2.74b	100.11 ± 2.44b	0.47 ± 0.01b	1.82 ± 0.08b
		RT	145.00 ± 3.12a	227.45 ± 3.61a	0.70 ± 0.02a	2.92 ± 0.09a
		SR	136.00 ± 2.75a	221.42 ± 2.05a	0.66 ± 0.01a	2.69 ± 0.06a
		SB	139.33 ± 2.54a	226.55 ± 2.76a	0.72 ± 0.02a	2.76 ± 0.07a
Wheat	AOA	CK	65.67 ± 1.01b	118.40 ± 3.12b	0.71 ± 0.01b	3.21 ± 0.23b
		RT	73.00 ± 0.84a	129.35 ± 2.84a	0.87 ± 0.02a	3.90 ± 0.54a
		SR	72.00 ± 0.92a	130.18 ± 1.74a	0.90 ± 0.01a	4.14 ± 0.27a
		SB	69.00 ± 1.12a	122.10 ± 2.03a	0.89 ± 0.01a	4.09 ± 0.36a
	AOB	CK	192.36 ± 8.74d	195.14 ± 7.23d	0.61 ± 0.01d	2.79 ± 0.07d
		RT	286.79 ± 9.22c	301.95 ± 10.74c	0.75 ± 0.02c	3.24 ± 0.09c
		SR	318.02 ± 7.91b	346.00 ± 11.95b	0.80 ± 0.02b	3.53 ± 0.10b
		SB	334.96 ± 10.35a	363.61 ± 12.02a	0.84 ± 0.02a	3.71 ± 0.11a

Dates in the table are Mean ± SE; Different letters in the same column indicate a significant difference (*p* < 0.05).

### Community composition of AOA and AOB

**Figure 3:**
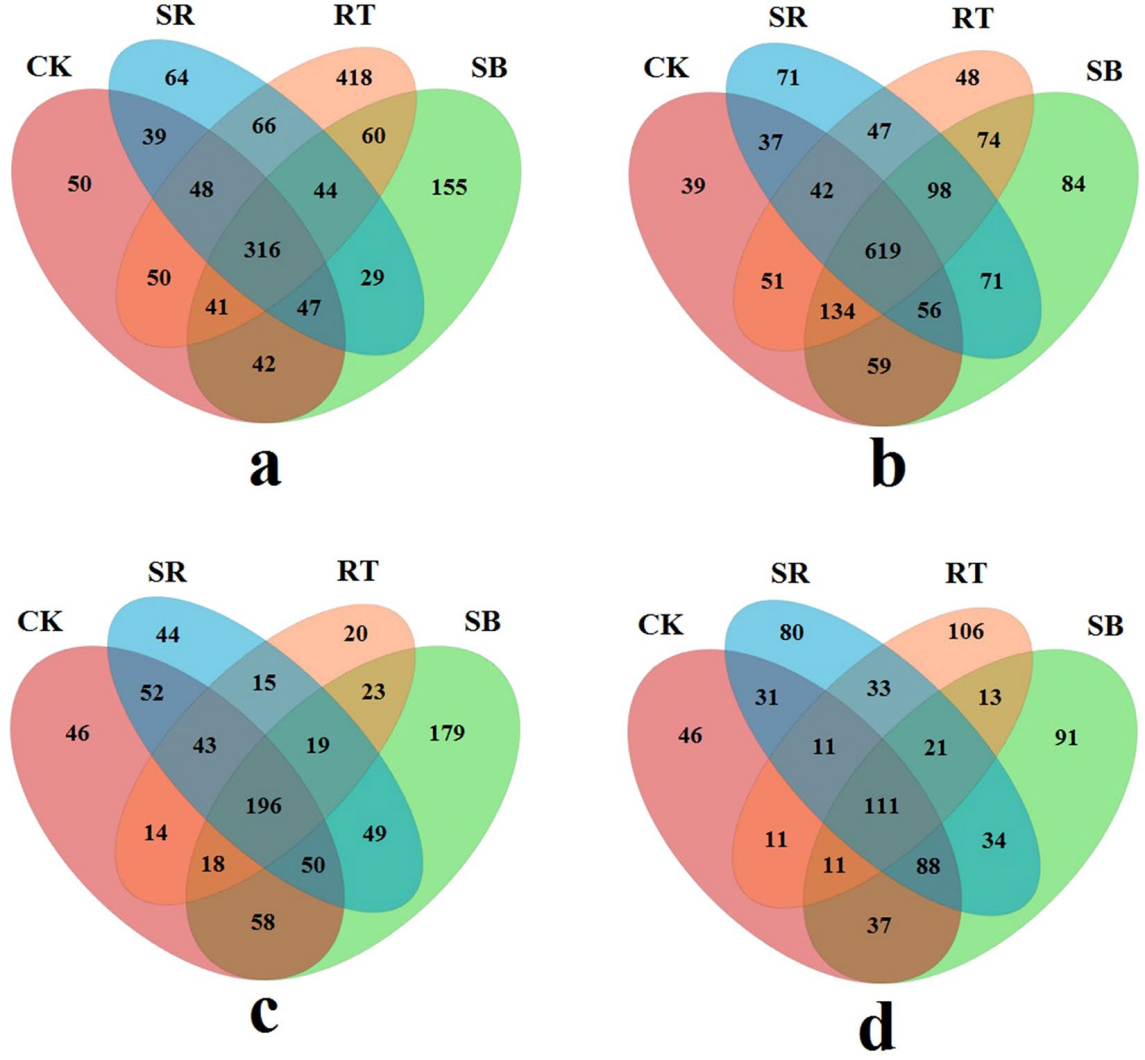
Venn diagrams of OTU richness of AOA (**a** for rice season, **b** for wheat season) and AOB (**c** for rice season, **d** for wheat season).

Shared and unique genera among 4 fertilization regimes were determined via Venn diagram (Fig. 3). A total of 1314 AOA-related OTUs and 1530 AOB-related OTUs under 4 treatments were observed in the rice season, respectively. The numbers of shared AOA and AOB related OTUs were 316 (24.0% of total) and 619 (40.5% of total), respectively. A total of 826 AOA-related OTUs and 724 AOB-related OTUs were observed in the wheat season, respectively. The numbers of shared AOA and AOB related OTUs were 196 (23.7% of total) and 111 (15.3% of total), respectively.

**Figure 4:**
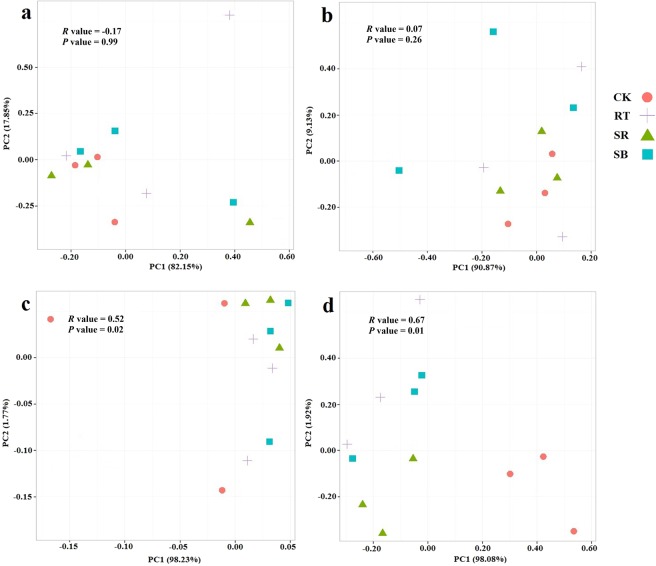
Principle component analysis (PCA) of AOA (**a** for rice season, **b** for wheat season) and AOB (**c** for rice season, **d** for wheat season).

PCA analysis showed that there were no group effects on the composition of AOA communities among 4 treatments in both rice and wheat season (Fig. 4). However, the composition of AOB communities under CK and other 3 fertilization treatments were separated by the first component (PC1) in rice and wheat season, which explained 98.23% and 98.08% of variation, respectively. In the wheat season, the AOB community composition under SR and RT were separated by the second component (PC2), explaining 1.92% of variation. It was also confirmed by ANOSIM that the *R* values of AOB composition (*R* value = 0.52 and 0.67, *P* value = 0.02 and 0.01 in rice and wheat season, respectively) were the higher than which of AOA composition. The results showed that fertilization regimes exerted higher influence on the composition of AOB community than on that of AOA. Among the treatments of RT, SR and SB, there was only one significant difference between SR and RT in AOB community composition of winter season (*R* value = 0.58, *P* value = 0.02).

### Correlation between soil properties and abundances of AOA and AOB

Pearson’s correlation was applied to determine the relationships between soil properties and abundances of AOA and AOB (Table 3). In the rice season, the AOA abundance was significantly and negatively correlated to pH (*P* < 0.01) and C/N (*P* < 0.05). The AOB abundance was significantly and negatively correlated to pH (*P* < 0.01) and positively correlated to exchangeable NH_4_^+^-N (*P* < 0.01). In the wheat season, the AOA abundance was significantly and negatively correlated to pH (*P* < 0.01), while positively correlated to SOC (*P* < 0.01). The AOB abundance was significantly and negatively correlated to pH (*P* < 0.01) and also positively correlated to SOC (*P* < 0.01).

**Table 3:** Correlation analysis of soil properties and amoA gene abundances of AOA and AOB.

Seasons		pH	Bulk density	SOC	TN	NO_3_^−^-N	NH_4_^+^-N	C/N
Rice	AOA	−0.64**	−0.09	0.41	0.39	0.47	0.32	−0.56*
	AOB	−0.43**	−0.15	0.35	0.32	0.38	0.46**	−0.18
Wheat	AOA	−0.60**	−0.39	0.72**	0.39	0.37	0.36	0.53
	AOB	−0.73**	−0.57	0.79**	0.52	0.41	0.60*	0.54

Abbreviations: SOC, soil organic C; TN, total nitrogen.

*Indicate significant correlations at *P* < 0.05, **Indicate significant correlations at *P* < 0.01.

### Soil potential nitrification rate (PNR) and relative contributions of AOA and AOB to nitrification

**Figure 5:**
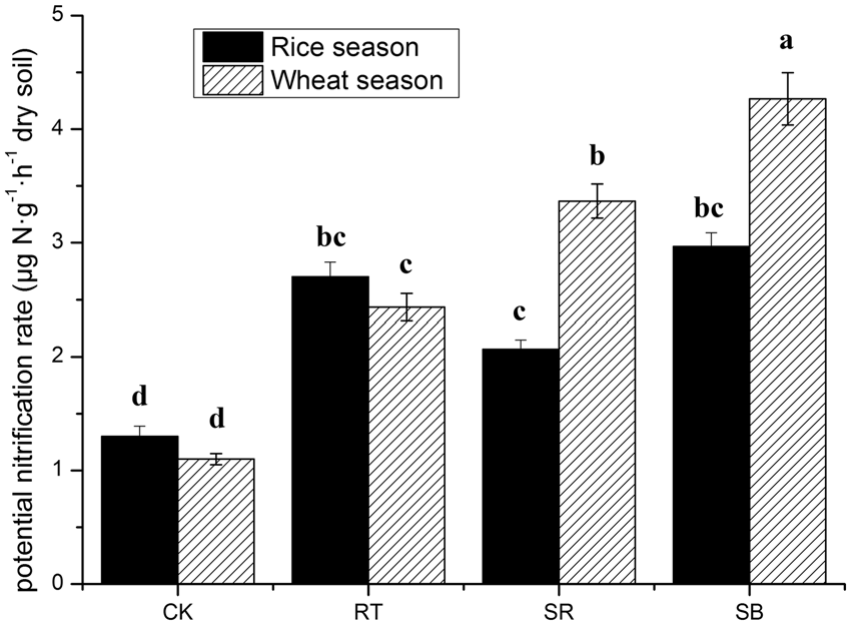
The potential nitrification rates (PNR) in harvest stage of rice and wheat season. Different letters indicate a significant difference (*P* < 0.05).

**Figure 6:**
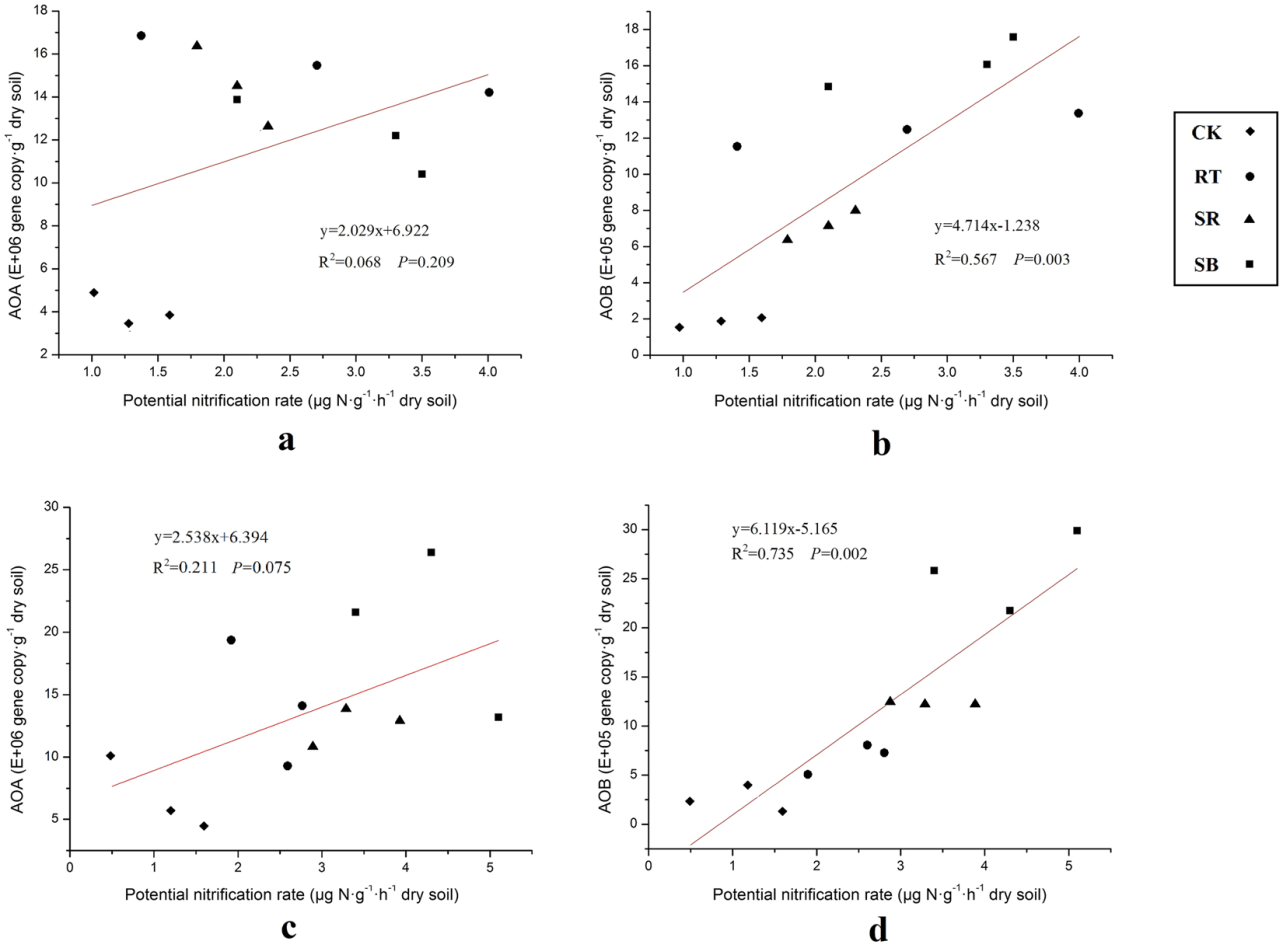
Correlation analysis between potential nitrification rate (PNR) and AOA (**a** for rice season, **b** for wheat season) and AOB (**c** for rice season, **d** for wheat season) gene copy abundances.

Soil PNR under 4 treatments ranged from 1.30–2.97 and 1.10–4.27 μg N·g^−1^·h^−1^ dry soil in rice and wheat season, respectively (Fig. 5). PNR of treatments with fertilization were greater than that of CK in the whole year. There was no significant difference of PNR among RT, SR and SB in the rice season. On the contrary, PNR in the wheat season among RT, SR and SB were significantly different. The sequence was SB > SR > RT (*P* < 0.05). PNR was not significantly correlated with AOA abundance in rice (R^2^ = 0.068, *P* = 0.209) and wheat season (R^2^ = 0.211, *P* = 0.075) (Fig. 6). However, PNR was positively correlated with AOB abundances both in rice (R^2^ = 0.567, *P* = 0.003) and wheat season (R^2^ = 0.735, *P* = 0.002).

## Discussion

### AOB and AOA abundance and community diversity response to straw and straw biochar

In both acid and alkaline soil, AOA outnumbered AOB in most cases irrespective of crop types, fertilization and irrigation managements, and AOA/AOB ratio varied from 1.2 to 2383 in the bulk and rhizosphere soil^21,27–29^. Only a few exceptions found that AOB abundance was higher than AOA (AOA/AOB ratio ranged from 0.39 to 12.3)^30–32^. The result of present study confirmed that amoA gene numbers of AOA were greater than those of AOB in the rice-wheat rotation system under different fertilization regimes.

Our study showed that compared to RT and SR, SB stimulated the growth of AOA and AOB in the wheat season, but there was no significant difference among the fertilization treatments in the rice season. Song^13^ found that the addition of biochar derived from cotton stalk increased the abundance of AOA and AOB in coastal alkaline soil and the same increase by wood biochar was reported by Prommer^33^. Bi^19^ reported that the combination of rice straw biochar with N fertilizer could increase AOB abundance significantly in greenhouse vegetable soil, but had no effect on AOA abundance. He^20^ suggested that biochar increased AOA and AOB abundances in oxisols (acid soil), but not in cambosols (alkaline soil). The inconsistency indicated the effects of biochar on the abundance of ammonia oxidizers may be influenced by soil pH (acid soil or alkaline soil), crop type (grain fields or vegetable fields), oxygen level (flooded soil or dry soil), etc, and it is difficult to draw a general conclusion.

Compared to RT, straw + N fertilizer increased AOB abundance significantly in the wheat season, while had no influences on AOB abundance in the rice season and AOA abundance in the whole year. Similar results were reported by Hai^17^ and Lin^33^. However, Wessén^34^ observed that there was no change of AOA and AOB amoA gene numbers under the treatment of maize straw + N fertilizer in an acido-neutral soil condition (pH ranged from 5.6–6.6). AOB was widely considered to be favorable in alkaline and neutral soil^35,36^. The reason for AOB abundance increase in our study may be because of the higher soil pH (pH ranged from 8.0–8.6 in our study, and 5.6–6.6 in reference), which could generate more NH_3_ than NH_4_^+^ as the substrate, and was more beneficial for the growing of AOB^37^.

The abundances of ammonia oxidizers were also highly dependent on soil properties, which were strongly influenced by fertilization regimes. Soil pH was considered as a vital environmental factor to influence ammonia-oxidizing bacterial and archaeal community. Extensive studies demonstrated that soil pH (ranged from 3.5–8.7, including acidic, acido-neutral, and alkalinophilic conditions) was strongly correlated with AOB and AOA abundances^37–39^. Our results were similar to these previous studies that Pearson’s correlation analysis showed that AOB and AOA abundances increased significantly with the decrease of soil pH (*P* < 0.05) in both rice and wheat season (Table 3). Compared to RT, SR and SB decreased soil pH significantly (Table 1). Therefore, the additions of straw and straw biochar had the potential to enhance AOB and AOA abundances. In our study, the growth AOB was highly stimulated when pH decreased in wheat season, while the response of AOA abundance was not significant. The reason for little increase of AOA abundance (Fig. 2) may also attribute to soil pH. AOB prefer to grow in neutral and alkaline soil, but the growth of AOA was considered more suitable in acid environment, and the response of AOA to environmental factors was weak in alkaline soil^5,27,28^.

Exchangeable NH_4_^+^-N was a direct substrate resource for ammonia oxidizers, and the increase of exchangeable NH_4_^+^-N concentration often led to a significant impact on AOB abundance^31,40^. Correlation analysis in our study had similar results that NH_4_^+^-N concentration was positive to AOB abundance in rice (*P* < 0.01) and wheat season (*P* < 0.05), while there was no clear correlation to the AOA abundance. After 7 years cultivation, the addition of straw and straw biochar increased exchangeable NH_4_^+^-N concentration in the rice season, showing the potential to stimulate AOB growing.

SOC was another environmental factor that influenced ammonia oxidizers abundances significantly. AOA and AOB abundances were both positive to SOC in the wheat season (*P* < 0.01). These findings were agreed with several previous studies who reported AOA and AOB abundances increased with SOC in dry land, such as cotton, maize^31,41^. Besides, the AOA abundance was negatively correlated with C/N in the rice season (*P* < 0.05). Extensive researches suggested that AOA could withstand some extreme environment, like low oxygen level as in rice season, and also had the ability to be alternative between chemoautotrophy and heterotrophic growth according to different carbon supplying conditions^42–44^. Therefore, the ability to metabolize other energy sources like rice root exudates under anaerobic condition may lead to the negative correlation between AOA abundance and C/N.

According to the PCA analysis (Fig. 4), 3 fertilized treatments were distinctly separated from unfertilized CK in rice and wheat season, suggesting long-term N input influenced AOB community in the rice-wheat rotation system. This was agreed to some previous studies in both dry (cotton field) and wet agricultural soil (paddy field), found distinct differences in AOB community composition between fertilized and unfertilized soil^21,31,45^. Among three fertilized treatments, there was only one significant difference between SR and RT in AOB community composition of winter season (*R* value = 0.58, *P* value = 0.02), indicating the higher effect of straw addition than straw biochar on AOB community composition. Conversely, there was no difference about AOA community between 4 treatments in a whole year. However, there was no consensus about the effects of fertilization regimes on AOA community. Some researchers reported AOA composition was separated between fertilized and unfertilized treatments^4,34^; while some revealed AOA abundance and structure remained constant after fertilization^46,47^. The effects were varied from different agricultural management, climate and geographic conditions. This inconsistency of the effects on AOB and AOA may due to the soil nutrient level. Several previous studies found AOB was more favorable in nitrogen rich soil while AOA prefer low nitrogen level soil^48,49^.

Based on the alpha-diversity analysis (Table 2), our findings evidenced that for both AOA and AOB, fertilized treatments were all higher than CK in the whole year. For all the indices estimated, the sequence of AOB diversity was SB > SR > RT (*P* < 0.05) in the wheat season. There were generally three reasons causing the effects of biochar and straw on the diversity of AOB. The first was the input of organic C and N through the addition of biochar and straw, which could directly provide substantial nutrient for AOB^36,37^; the second was the addition of organic improved soil properties (Table 1), such as balancing pH, reducing bulk density etc, enhancing AOB community diversity; the last was the porous structure of biochar, which was beneficial to soil water and nutrient retention, strengthening the sustainability of nutrient supply^50^.

Venn diagram (Fig. 3) demonstrated that in rice and wheat season, the shared AOA-related OTUs were 24.0% and 23.7% of total, while the shared AOB-related OTUs were 40.5% and 15.3% of total, respectively. These findings suggested fertilization regimes affected AOA-related OTUs almost equally in rice and wheat season, while the influence of fertilization on AOB-related OTUs in the wheat season was much more than in rice season. AOA and AOB are normally aerobic microbes. The activity of AOB might be depressed in the rice season due to the lower oxygen level caused by flooded soil environment^21,33^. However, AOA could keep active in the rice season because of the characters to be adapted to a wider range of oxygen level and the ability to switch between autotrophy and heterotrophy^42,44^.

### Relative contributions of AOA and AOB to nitrification

Fertilization could increase agricultural soil PNR significantly. In the present study, no significant difference of PNR was observed among different fertilization regimes in the rice season, while the situation was opposite in the wheat season. PNR of organic addition treatments were higher than SR, and SB was the highest treatment (*P* < 0.05). This was partially supporting some recent reports, which evidenced that synthetic fertilizer combined with biochar, cattle manure or organic fertilizer enhanced soil PNR in dry agricultural soil^9,19,31^. Organic addition might have the ability to stimulate soil nitrification and enhance the available nutrient supply for crops.

Although AOA outnumbered AOB in the whole year, the results of regression analysis demonstrated that PNR was highly correlated with AOB abundance in both rice and wheat season (*P* < 0.01), not AOA abundance (Fig. 6). Wang^9^ tested in 8 periods in one rice-wheat rotation, and found AOB was more important than AOA in ammonia oxidation process. Our results also were similar to Jia^51^ and Di^35^, who reported nitrification was driven by AOB rather than AOA. It’s worth noting that these patterns were all observed in alkaline or neutral and nitrogen-rich agricultural soil. Lower correlation with PNR should not indicate the less importance of AOA in soil nitrification. Even in the AOB dominant situation, 10–20% of nitrification still was attributed to AOA^52^. AOA was also believed to play an important role in nitrification of acid soil or uncultivated land^53,54^. DNA Stable-Isotope Probing (DNA-SIP) should be applied in future experiment to further describe the specific contribution of AOA and AOB to nitrification.

Due to the characteristics of nutrients slow release of straw and straw biochar, after 7 years application, exchangeable NH_4_^+^-N under SR and SB were significantly higher than RT in the harvest stage of rice season (Table 1), which became an important stimulation of AOB growth in wheat season, leading to the higher abundance and diversity of AOB community^52,55^. This could also be the reason for the rise of wheat yield (Fig. 1). Therefore, under the alkaline soil condition, controlling the activity of AOB in rice-wheat rotation system through agricultural management or NH_4_^+^-N addition would be an effective potential strategy to adjust soil nitrification intensity and therefore to improve the crop yield.

In this study, qPCR and high throughout pyrosequencing were employed to evaluate the responses of ammonia oxidizers to successive straw and straw biochar addition in alkaline rice-wheat rotation soil for 7 years. Our results demonstrated straw and straw biochar addition affected AOB more than AOA, especially in the wheat season. Compared to regular fertilization, straw and straw biochar addition increased AOB abundance and diversity significantly only in wheat season (*P* < 0.05), and they both ranked as SB > SR > RT. Among fertilized treatments, a significant difference between SR and RT was found in AOB community composition of the winter season (*R* value = 0.58, *P* value = 0.02). However, AOA was less responsive to organic addition, except the enhancement of abundance by biochar in the wheat season. The abundance of AOB and AOA was negatively correlated with soil pH both in rice and wheat season (*P* < 0.01), and positively correlated with SOC only in wheat season (*P* < 0.01). Under our experimental alkaline and nitrogen-rich soil conditions, straw and straw biochar increased soil PNR significantly. AOB abundance was highly positive to soil PNR in both rice and wheat season (*P* < 0.01), not to AOA abundance, indicating that with the addition of straw and straw biochar, the soil ammonia oxidation process was primarily driven by AOB rather than AOA. AOB had the potential to be regulated for better nitrogen uptake and crop yield in the rice-wheat rotation system.
